# Polypyrrole-Barium Ferrite Magnetic Cryogels for Water Purification

**DOI:** 10.3390/gels9020092

**Published:** 2023-01-20

**Authors:** Konstantin A. Milakin, Oumayma Taboubi, Udit Acharya, Miloslav Lhotka, Václav Pokorný, Magdalena Konefał, Olga Kočková, Jiřina Hromádková, Jiří Hodan, Patrycja Bober

**Affiliations:** 1 Institute of Macromolecular Chemistry, Czech Academy of Sciences, 162 06 Prague, Czech Republic; 2 Faculty of Chemical Technology, University of Chemistry and Technology Prague, 166 28 Prague, Czech Republic

**Keywords:** polypyrrole, barium ferrite, cryogel, dye, adsorption

## Abstract

Magnetic polypyrrole-gelatin-barium ferrite (PPy-G-BaFe) cryogels/aerogels were synthesized by one-step oxidative cryopolymerization of pyrrole in the presence of various fractions of barium ferrite (BaFe) nanoparticles, dispersed in aqueous gelatin solution. The successful incorporation of BaFe into the composites was confirmed by elemental analysis and scanning electron microscopy paired with an energy-dispersive X-ray detector. The maximum achieved content of BaFe in the resulting material was 3.9 wt%. The aerogels with incorporated BaFe had significantly higher specific surface area and conductivity, reaching 19.3 m^2^ g^−1^ and 4 × 10^−4^ S cm^−1^, respectively, compared to PPy-G aerogel, prepared in the absence of BaFe (7.3 m^2^ g^−1^ and 1 × 10^−5^ S cm^−1^). The model adsorption experiment using an anionic dye, Reactive Black 5, showed that magnetic PPy-G-BaFe aerogel, prepared at 10 wt% BaFe fraction, had significantly higher adsorption rate and higher adsorption capacity, compared to PPy-G (dye removal fraction 99.6% and 89.1%, respectively, after 23 h). Therefore, the prepared PPy-G-BaFe aerogels are attractive adsorbents for water purification due to their enhanced adsorption performance and the possibility of facilitated separation from solution by a magnetic field.

## 1. Introduction

Providing the world with clean and accessible water is a global challenge that has been affecting the everyday life of billions of people [1]. Development of technology and industry, utilizing fresh water supplies and producing wastewater, which is often contaminated and has to be purified before further use, contributes to this problem. Organic dyes are one of the most common contaminants of wastewater discharged by the facilities in the textile, leather or paper industries [2,3,4]. The mentioned contaminants can cause serious adverse health effects due to their potential mutagenicity and carcinogenicity [4], and therefore, the development of new methods and materials for water purification is an important issue.

Adsorption is one of the most effective and simple methods for the removal of water contaminants [5]. It uses various classes of adsorbents, including carbon-based materials [6], clays [7], zeolites [8], metal-organic frameworks [9] and polymers [10]. Conducting polymers have recently drawn attention as prospective materials for adsorption [2] due to their flexibility in chemical structure modification, which allows tuning for specific adsorbates and their responsiveness to various stimuli, which can be utilized for controlling the adsorption/desorption process.

Polypyrrole (PPy) is one of conducting polymers that have been widely used for the adsorption of dyes for water purification [11,12,13,14,15]. As a result of the conventional chemical oxidative polymerization, PPy is obtained as a powder [16], and, therefore, it is beneficial to develop other forms of PPy-based materials to facilitate their separation from the medium after purification. PPy-based cryogels can serve as a prospective material for this purpose due to their high mechanical stability and macroporous structure with high specific surface area [17]. They can be prepared by a facile one-step approach involving oxidative cryopolymerization of pyrrole in the frozen aqueous solution of a polymer, such as gelatin [17] or poly(vinyl alcohol) [18], which serves as a stabilizer for enhancing the mechanical properties of the resulting cryogel.

In order to further facilitate the separation of PPy-based materials from the treated solution after adsorption, magnetic particles can be incorporated into the composites, and such magnetic adsorbents have been reported before [19,20,21]. Moreover, magnetic cryogels, based on another type of conducting polymer, polyaniline, have also been used for the adsorption of dyes in aqueous media [22]. However, to the best of our knowledge, PPy cryogels have not been yet applied for this purpose. It can be beneficial to use them over polyaniline-based materials, for example, for the adsorption of anionic dyes. PPy (pK_a_ 9–11 [23]) has higher pK_a_ than polyaniline (pK_a_ ~3 [24]), which can allow more flexibility in adsorption conditions, such as a wider pH window. Therefore, in the present work, novel polypyrrole-gelatin (PPy-G) cryogels with incorporated magnetic barium ferrite (BaFe) nanoparticles have been prepared, characterized, and the possibility of their potential application for dye adsorption from water solutions has been assessed. Reactive Black 5, the anionic azo dye, one of the most used dyes in the textile industry for cotton and other cellulose fibers [25,26], was chosen as a model compound for adsorption experiments. The proposed combination of PPy, gelatin and BaFe in the composite can potentially lead to synergistic enhancement of adsorption performance towards Reactive Black 5. It has been shown that gelatin type A, used in this work, can be successfully utilized for the adsorption of anionic dyes [27] due to it being positively charged in neutral and acidic conditions [28]. BaFe nanoparticles can also contribute to dye adsorption by providing surface OH-groups, which can interact with the dyes by hydrogen bonding [29].

## 2. Results and Discussion

PPy-G-BaFe cryogels were synthesized by oxidative cryopolymerization of pyrrole in the presence of BaFe particles suspended in an aqueous gelatin solution. The polymerization conditions for the incorporation of the magnetic particles were chosen based on our previous work [17], which shows that 6 wt% of gelatin is the optimal concentration for the preparation of mechanically stable PPy-G cryogel.

### 2.1. Material Morphology and Composition

**Figure 1:**
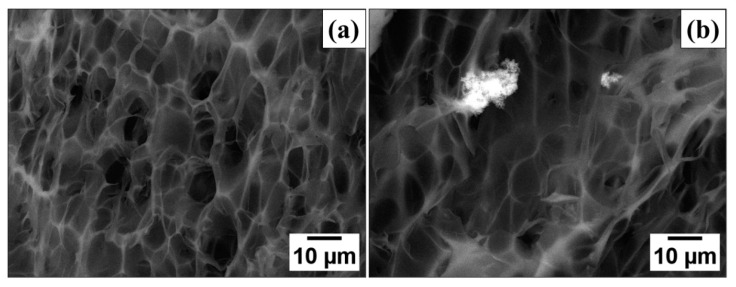
SEM/BSE images of (**a**) PPy-G aerogel, prepared in the absence of BaFe and (**b**) PPy-G-BaFe aerogel, synthesized using 10 wt% of BaFe.

SEM showed that the macroporous morphology (pore size < 30 μm), which is expected for the cryogels/aerogels, was obtained for all prepared PPy-G-BaFe materials. Due to the similarity of the obtained SEM images, only the ones for PPy-G aerogel, prepared in the absence of BaFe, and PPy-G-BaFe aerogel, synthesized using 10 wt% of BaFe are provided in Figure 1 (SEM images for the composites, prepared using the other BaFe fractions are given in Supporting Information (Figure S1). BSE imaging confirms the successful incorporation of BaFe particles into PPy-G-BaFe aerogels, showing them as bright spots in the image (Figure 1b).

SEM/EDX (Table 1) measurement was additionally performed to confirm the successful incorporation of BaFe particles into PPy-G-BaFe aerogels. It showed the presence of barium and iron atoms in the materials, which were absent in PPy-G aerogels prepared without BaFe. It should be noted that the values of iron content, determined by SEM/EDX and provided in Table 1, are only for qualitative proof of the presence of the magnetic particles in the obtained composites. They do not represent the quantitative composition of the bulk of the materials due to the sensitivity of the method to the local distribution of the phases.

Elemental analysis was used for further confirmation of BaFe presence in PPy-G-BaFe aerogels. As can be seen from Table 1, the increase of the BaFe particle fraction in the polymerization mixture corresponds to the higher barium content in the resulting material. The recalculation of the measured barium fraction into the fraction of barium ferrite showed that the maximum achieved amount of the magnetic particles in the final material was 3.9 wt%.

### 2.2. Material Properties

The presence of BaFe nanoparticles in the cryogels does not significantly affect their tensile characteristics, as can be seen in Table 2. The mechanical properties of the materials are mainly determined by the PPy-G cryogel matrix, which shows good mechanical stability, which is important for water purification applications.

Specific surface area is another crucial property of the adsorbents. Table 2 shows that increasing BaFe content in PPy-G-BaFe aerogels leads to an increase in the specific surface area of the materials. The highest value (19.3 m^2^ g^−1^) was achieved for PPy-G-BaFe aerogel, synthesized using 10 wt% BaFe, which is ≈3 times higher, compared to PPy-G aerogel (7.3 m^2^ g^−1^). The specific surface area of pristine BaFe nanoparticles was found to be 15.0 m^2^ g^−1^.

As can be seen from Table 2, the introduction of barium ferrite particles into PPy-G aerogels led to the increase of conductivity by about 1 order of magnitude. The highest conductivity 4 × 10^−4^ S cm^−1^ was achieved for the aerogel, synthesized at 5 wt% BaFe fraction, while the value for PPy-G aerogel, prepared in the absence of BaFe, was 1 × 10^−5^ S cm^−1^. Similar behavior has also been observed for the other conducting polymer-metal oxide composites and has been attributed to the change in the conducting polymer chain packing induced by the incorporation of metal oxide particles [30].

### 2.3. Spectroscopy

**Figure 2:**
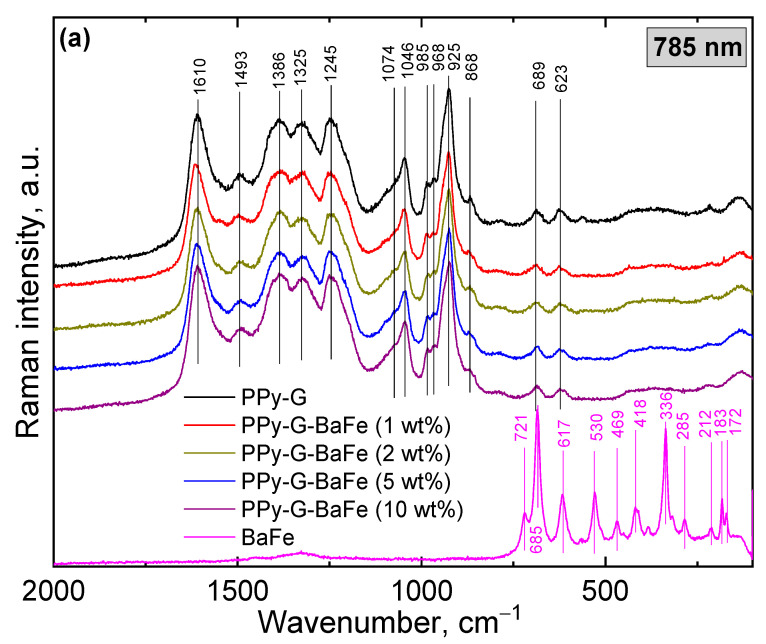

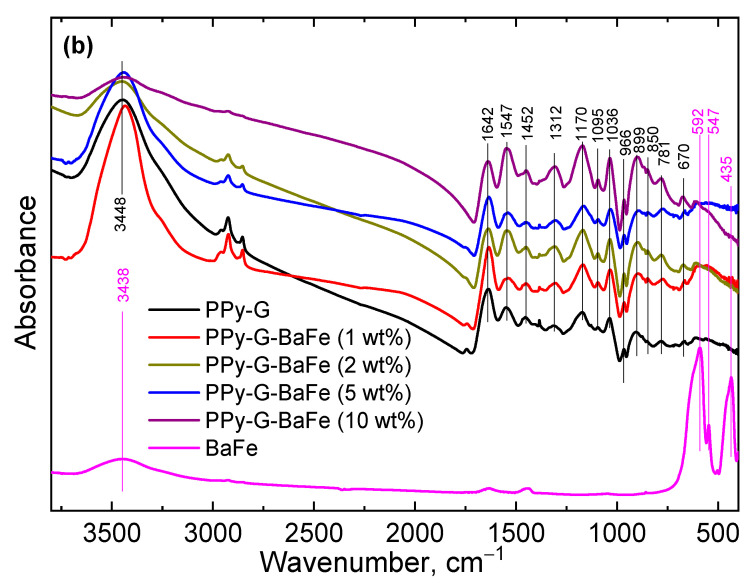
(**a**) Raman spectra (exc. 785 nm) and (**b**) FTIR spectra of PPy-G aerogel, pristine BaFe and PPy-G-BaFe aerogels, prepared using various BaFe fractions. The solid black lines refer to PPy-G aerogel peaks. The magenta lines refer to BaFe bands.

The Raman and FTIR spectra of pristine BaFe, PPy-G aerogel and PPy-G-BaFe aerogels obtained at various BaFe fractions are depicted in Figure 2. In the Raman spectrum of barium ferrite (Figure 2a), two prominent Raman modes at 685 cm^−1^ (assigned to the motion of the FeO_5_ bipyramidal group (A_1g_)) and 336 cm^−1^ (referred to octahedral (12 k) due to (E_2g_) symmetry in the BaFe_12_O_19_ structure) are well-distinguished [31,32,33,34,35]. The FTIR spectrum of BaFe (Figure 2b) shows mainly three bands: 592, 547 and 435 cm^−1^, which correspond to the symmetric stretching of (Fe-O) and a broad band at 3438 cm^−1^ attributed to the vibration of stretching of the O-H bond associated with the adsorbed water [31,32,33].

The successful synthesis of PPy by cryopolymerization is proven by both spectroscopic methods. Raman and FTIR spectra of PPy-G and PPy-G-BaFe aerogels, obtained at various BaFe fractions, are mostly identical and show the characteristic bands of PPy as reported previously in the literature [36,37,38]. Based on the other characterization methods, BaFe is embedded within the composite aerogels, but the presence of interaction between BaFe and PPy cannot be estimated from the spectroscopy data. The presence of gelatin is confirmed in Figure 2b with the band at 1642 cm^−1^ (amide I related to the C=O stretching vibrations coupled with COO^−^) [36]. However, this band is not detected in the Raman spectra as the PPy signal is resonantly enhanced with a 785 nm excitation line [17].

**Figure 3:**
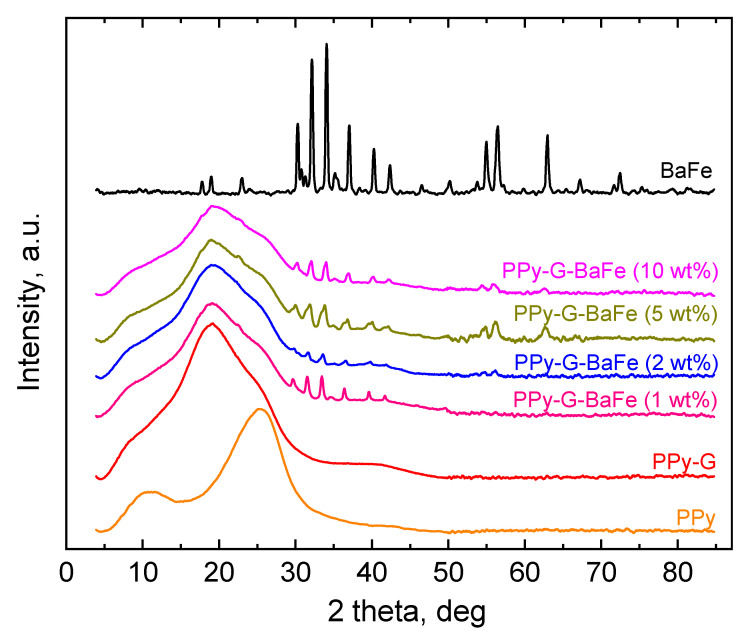
XRD patterns of PPy-G-BaFe aerogels prepared using various fractions of BaFe, PPy-G aerogel, pristine PPy and pristine BaFe.

XRD patterns of PPy-G-BaFe aerogels, prepared using various BaFe fractions, PPy-G aerogel, pristine PPy and pristine BaFe are presented in Figure 3. The well-defined peaks observed in the XRD pattern of pristine BaFe, are in agreement with the characteristic peaks of the hexagonal barium ferrite phase [39]. The XRD pattern of PPy-G aerogel is characterized by a wide peak around 19°. Peak deconvolution of the scattering profile in this region reveals three broad peaks, as can be seen in Figure S2. The peaks at 11.4° and 25.2° are typical for PPy, while the 19.2° peak originates from gelatin. Broad peaks on the XRD spectra could reveal some structural ordering of the nanometric scale, which is characteristic of nanostructured polymers with a low degree of crystallinity [40]. The 25.2° peak may be attributed to the scattering from PPy chains at the interplanar spacing [41].

The XRD spectra of all PPy-G-BaFe composites are comprised of both a broad peak from PPy-G contribution and the diffraction peaks from pristine BaFe. With the increasing amount of BaFe, the intensity of the PPy-G peak decreases, which suggests that the presence of BaFe has influenced the organization of the structure of the PPy-G phase. It could be expected that the diffraction peaks of BaFe should become more distinguishable with increasing ferrite content; however, the spectra do not follow this pattern. It could imply that the structure of the composite is not homogenous.

The BaFe peaks in the composites are also slightly shifted towards lower 2Θ values compared to the spectrum of pristine BaFe (Figure S3). Such a shift was observed before in the literature for (barium ferrite/silica)@PPy [42] and polyaniline-hexaferrite [43] composites. It has been attributed to either some specific interactions between BaFe and PPy [42] or distortion (stretching) of hexaferrite structure [43], which is presumably occurring due to the polymer penetration inside the hexaferrite particles. In our case, the mentioned peak shift is the highest for PPy-G-BaFe aerogel, prepared at 1 wt% BaFe fraction, and it gradually decreases with increasing the ferrite content in the material. Changing the BaFe:PPy ratio can affect both the degree of interaction and degree of PPy penetration inside the ferrite particles and the consequent distortion of their structure. Both factors are expected to be the most pronounced for the material with the lowest content of BaFe, leading to the highest peak shift.

### 2.4. Dye Adsorption

**Figure 4:**
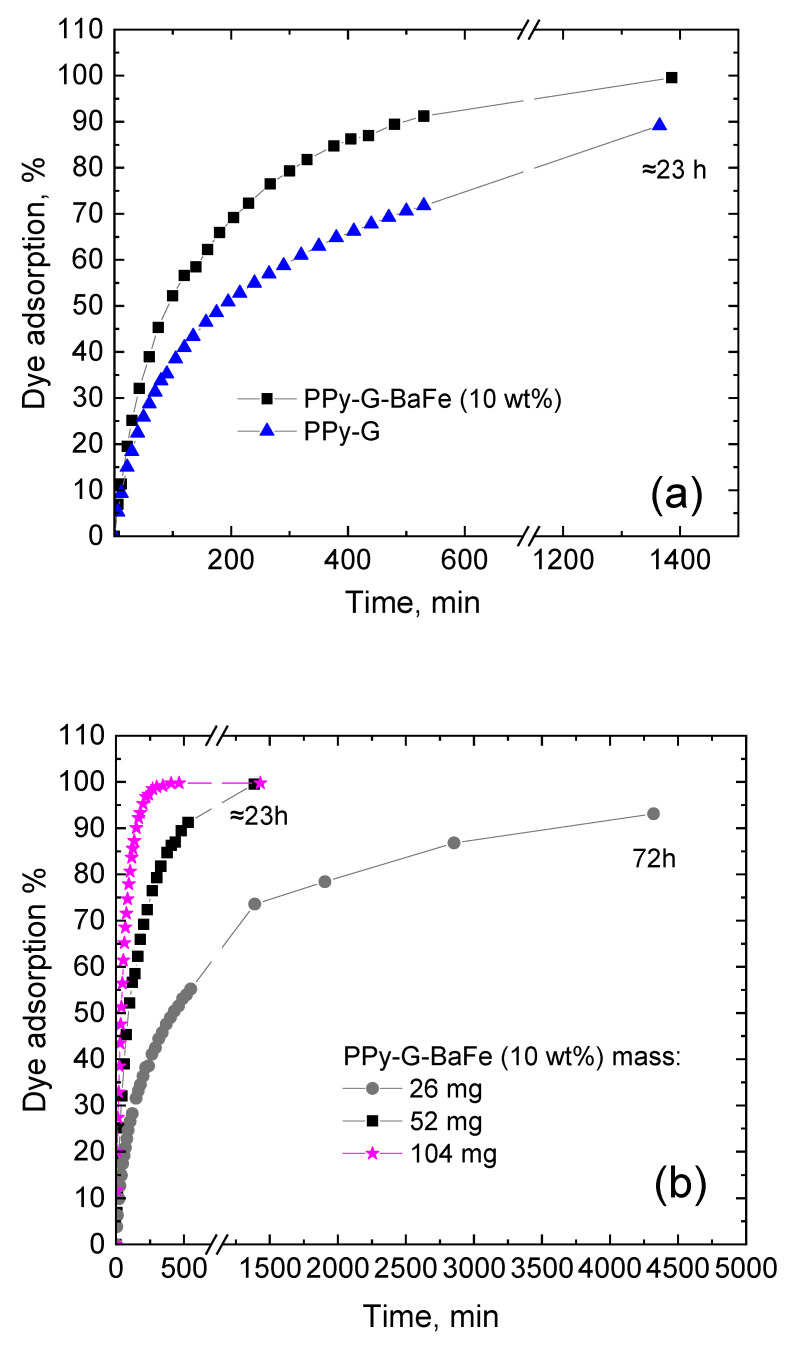
Adsorption of Reactive Black 5 over time by (**a**) 52 mg PPy-G-BaFe aerogel (prepared at the 10 wt% BaFe fraction) and PPy-G aerogel, and (**b**) 26 and 104 mg of PPy-G-BaFe aerogel, prepared at the 10 wt% BaFe fraction.

**Figure 5:**
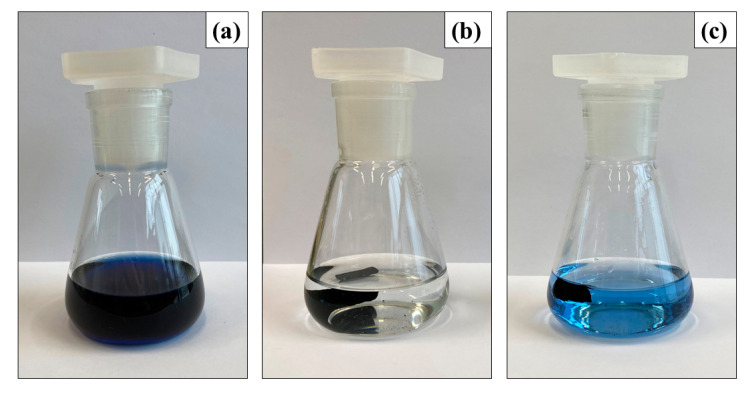
Photos of Reactive Black 5 solutions (**a**) before adsorption and after ≈23 h of adsorption with 52 mg of (**b**) PPy-G-BaFe aerogel, prepared at the 10 wt% BaFe fraction, and (**c**) PPy-G aerogel.

The possibility of application of the prepared PPy-G-BaFe aerogels as adsorbents for water purification was studied using a model anionic dye Reactive Black 5 (UV-vis spectra are provided in Supporting Information, Figure S4). It should be noted that the composites kept their mechanical integrity during the process, and the presence of magnetic particles can facilitate their separation from the treated solution. Figure 4a shows the comparison of adsorption kinetics (52 mg of adsorbent) of PPy-G-BaFe aerogel, prepared at the highest used BaFe fraction (10 wt%), and PPy-G aerogel. PPy-G-BaFe aerogel was found to have a significantly higher adsorption rate and higher adsorption capacity after ≈23 h compared to PPy-G. After ≈23 h, more than 99% of the dye was adsorbed in the presence of PPy-G-BaFe, while in the case of PPy-G, the value of the dye removal fraction was 89% (Figure 5). The difference is likely attributed to the higher specific surface area of the composite containing BaFe nanoparticles. Additionally, adsorption kinetics was studied for 26 and 104 mg of PPy-G-BaFe aerogel, prepared at 10 wt% BaFe fraction (Figure 4b). For the experiment with 26 mg of the adsorbent, 72 h was taken as the equilibrium point.

The obtained kinetics curves were adjusted to pseudo-first-order, pseudo-second-order and intraparticle diffusion models (Table 3). As can be seen from Table 3, the adsorption kinetics of Reactive Black 5 with PPy-G and PPy-G-BaFe as adsorbents follows the pseudo-second-order model, pointing at chemisorption being the rate-limiting step [44]. The adsorption performance of the prepared aerogels is comparable to the results previously reported for conducting polymer-based composites and other types of materials (Table S1).

## 3. Conclusions

Magnetic, microporous and mechanically stable PPy-G-BaFe cryogels/aerogels were prepared by one-step oxidative cryopolymerization. The aerogels with incorporated BaFe nanoparticles showed one order of magnitude higher conductivity and ≈3 times higher specific surface area compared to PPy-G aerogels prepared in the absence of BaFe. Due to their higher specific surface area, PPy-G-BaFe aerogels also showed higher adsorption capacity and adsorption rate in a model experiment with an anionic dye, Reactive Black 5. Thus, enhanced adsorption performance and the possibility of facilitated separation from the solution of magnetic PPy-G-BaFe aerogels makes them attractive materials for water purification application.

## 4. Materials and Methods

### 4.1. Materials and Preparation

**Figure 6:**
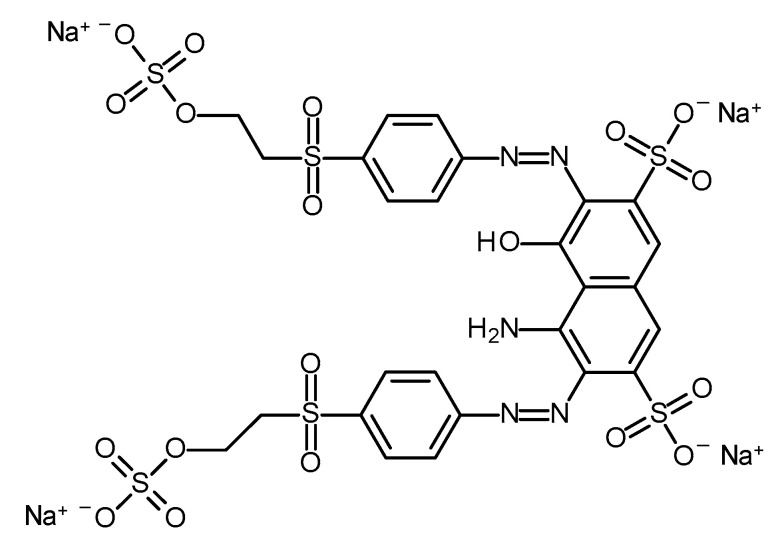
Chemical structure of Reactive Black 5.

Pyrrole (Sigma-Aldrich, Beijing, China), iron (III) chloride hexahydrate (Sigma-Aldrich, Taufkirchen, Germany), gelatin (from porcine skin, Sigma-Aldrich, Taufkirchen, Germany), barium ferrite (BaFe, BaFe_12_O_19_, nanopowder, <100 nm particle size, Sigma-Aldrich, St. Louis, MO, USA) and Reactive Black 5 (Sigma-Aldrich, Taufkirchen, Germany, Figure 6) were used as received.

Polypyrrole-gelatin-BaFe (PPy-G-BaFe) cryogels were prepared by oxidative cryopolymerization of pyrrole with FeCl_3_ in the presence of BaFe particles in an aqueous solution of gelatin (6 wt%). For the typical synthesis, 14 mmol of pyrrole and various amounts of BaFe nanoparticles were added into 35 mL of an aqueous solution of gelatin (6 wt%). The obtained suspension was sonicated for 30 min and subsequently left under mechanical stirring. The oxidant solution was prepared separately by the addition of 35 mmol of FeCl_3_·6H2O into 35 mL of 6 wt% gelatin solution in water. The prepared solutions, containing the oxidant and the monomer, were mixed under vigorous stirring, quickly sucked into plastic syringes, frozen in a dry ice/ethanol bath and left to polymerize in a freezer at –24 °C for 7 days. After the polymerization, the cryogels were allowed to thaw at room temperature, removed from the syringes, washed in excess of water and freeze-dried to obtain aerogels. The amount of BaFe, used in the synthesis was calculated based on the theoretical estimate of the PPy amount in the resulting material (1 g of pyrrole is converted to 1.3 g of PPy [45]). Thus, the fractions of BaFe were 1, 2, 5 and 10 wt% relative to PPy.

The PPy cryogel/aerogel with gelatin was prepared in the absence of BaFe by a similar procedure. It will be referred to as PPy-G cryogel/aerogel.

The neat PPy was prepared by a similar cryopolymerization method in the absence of gelatin and BaFe. After the polymerization, it was washed with water and freeze-dried.

### 4.2. Characterization

The morphology of aerogels was studied using a MAIA3 Tescan scanning electron microscope (SEM) in back-scattered (BSE) electron imaging mode. The energy-dispersive X-ray detector (EDX, X-MaxN 20, Oxford Instruments, Abingdon, UK) was utilized for the assessment of the elemental material composition.

Quantitative determination of barium content in the aerogels by elemental analysis was performed using an Inductively coupled plasma mass spectrometry (ICP-MS) instrument NexION 2000 B (Perkin Elmer, Waltham, MA, USA) after preliminary digestion of the samples in nitric acid. The amount of the sample adjusted according to the expected content of Ba (4–10 mg) was weighed into the glass vial, and 1 mL HNO_3_ was added. Digestion in the Microwave reactor Biotage Initiator (Biotage AB, Uppsala, Sweden) followed. After the digestion, a NexION 2000 B ICP-MS instrument was used for the quantitative determination of barium. A setup solution was used to calibrate the instrument (daily performance check). A standard solution of barium (c = 100 mg/L) in 5% HNO_3_ was used for the Ba calibration. The samples and standards were diluted to concentrations suitable for ICP-MS measurement with MilliQ purity water, prepared with the laboratory water purification system Milli-Q^®^ IQ 7000 (Merck KGaA, Darmstadt, Germany). The addition of 2.5% nitric acid (67–69%, Analpure Ultra, ANALYTIKA^®^ spol. s r.o., Prague, Czech Republic) stabilized the solutions. The concentration range for the calibration curve was from 0.01 μg/L to 1 μg/L.

Details of the instrument setup for the analysis are given below:

RF Power: 1100 watts; Plasma gas flow: 15 L/min; Auxiliary gas flow: 1.2 L/min; Nebulizer gas flow: 0.9 L/min; Nebulizer/spray chamber: Glass/Glass; Sampler/skimmer cones: nickel; Detector: Dual; Scanning mode: peak hopping; Dwell time: 50 ms; Integration time: 1000 ms; Number of points/peak: 1; Number of sweeps/readings: 20; Number of readings/replicates: 1; Number of replicates: 3.

The values of DC conductivity were obtained by using the van der Pauw method on pellets (diameter 13 mm), prepared at 70 kN using a hydraulic press Trystom H-62. A Keithley 230 Programmable Voltage Source connected in serial with a Keithley 196 System DMM served as a current source, while the potential difference was measured with a Keithley 181 Nanovoltmeter. The values were calculated as an average value from the measurements in two perpendicular directions at room temperature.

Equilibrium adsorption isotherms of nitrogen were measured at 77 K using a static volumetric adsorption system (TriFlex analyzer, Micrometrics, Ottawa, ON, Canada). The adsorption isotherms were fitted to obtain the Brunauer–Emmett–Teller (BET) specific surface area. Before the adsorption measurements, the samples were incubated at 40 °C for 48 h in a vacuum.

FTIR spectra of the powders dispersed in potassium bromide pellets were registered using a Thermo Nicolet NEXUS 870 FTIR spectrometer with a DTGS TEC detector in the wavenumber region 400–4000 cm^−1^.

Raman spectra excited with a near-infrared diode laser 785 nm were collected on a Renishaw InVia Reflex Raman microspectrometer. The scattered light was analyzed using a spectrograph with holographic grating 1200 lines mm^−1^. A research-grade Leica DM LM microscope was used to focus the laser beam. A Peltier-cooled CCD detector (576 × 384 pixels) registered the dispersed light.

Static mechanical properties of water-swollen cryogels (cylindrical shape–diameter 3 mm, length 60 mm) were studied using electromechanical testing machine Instron 6025/5800R equipped with a 10 N load cell at room temperature in deionized water and with a cross-head speed of 10 mm min^−1^. The presented values are averages of at least 3 measurements.

X-ray diffraction (XRD) patterns were collected using Bragg-Brentano and transmission geometries. The measurements in Bragg-Brentano geometry were performed under the CuKα radiation (wavelength λ = 1.54 Å) using a high-resolution GNR Explorer diffractometer with a Mythen 1 K strip detector, in the 2θ range of 4–85° with a step 0.1° and 10 s measurement time at each step. For transmission, the experiments were performed using a Rigaku pinhole camera (modified molecular metrology system) attached to a Rigaku MicroMax 003 micro-focused X-ray beam generator, operated at 50 kV and 0.6 mA (30 W). The camera was equipped with a vacuum-compatible version of the Pilatus3 R 300 K hybrid photon-counting detector. A sample-to-detector distance of 191.71 mm and an exposure time of 7200 s were used.

For the dye adsorption study, 52 mg of aerogels were immersed into an aqueous solution of Reactive Black (50 mg L^−1^, 50 mL) and kept under constant mechanical shaking (150 rpm) at room temperature in the dark. UV-vis spectra of the dye solution were collected at various time intervals without additional dilution using a Thermo Scientific Evolution 220 UV-vis spectrometer. Similar experiments were performed with 26 and 104 mg of PPy-G-BaFe (10 wt%) aerogels. For all the experiments, the equilibrium point was taken at ≈23 h (except the experiment with 26 mg of PPy-G-BaFe cryogel, for which it was 72 h). The dye removal fraction was calculated based on the following equation, where *A*_598,*t*_ and *A*_598,0_ are absorbance values of the peak at 598 nm at the defined time, *t*, and the initial absorbance, respectively:

$$\frac{A_{598,\;0}-A_{598,t}}{A_{598,0}}\times 100\%$$

The following equations were used for fitting the adsorption kinetics with the pseudo-first-order, pseudo-second-order and intraparticle diffusion models:

$$\mathrm{Pseudo}-\mathrm{first}-\mathrm{order}\;\mathrm{equation}:\;\log\left(Q_{e}–Q_{t}\right)=\log Q_{e}–\frac{k_{1}}{2.303}t$$

$$\mathrm{Pseudo}-\mathrm{second}-\mathrm{order}\;\mathrm{equation}:\;\frac{t}{Q_{t}}=\frac{1}{k_{2}Q_{e}^{2}}+\frac{t}{Q_{e}}$$

$$\mathrm{intraparticle}\;\mathrm{diffusion}\;\mathrm{equation}:\;Q_{t}=k_{i}t^{\frac{1}{2}}+c$$

where *Q_e_* and *Q_t_* (mg g^−1^) are the amounts of dye adsorbed at the equilibrium and the defined time, *t* (min), respectively, *k_1_* (min^−1^) is the pseudo-first-order rate constant, *k_2_* (g mg^−1^ min^−1^) is the pseudo-second-order rate constant, *k_i_* (mg g^−1^ min^−1/2^) is the intraparticle diffusion rate constant and *c* is the intraparticle diffusion constant.

*Q_e_* and *Q_t_* were calculated by the following equation:

$$Q_{e\left(t\right)}=\frac{\left(C_{0}-C_{e\left(t\right)}\right)}{m}\times V$$

where *C_0_* (mg L^−1^) is the initial dye concentration, *C_e(t)_* (mg L^−1^) is the dye concentration at the equilibrium (*e*) or the defined time (*t*), *m* (g) is the mass of the adsorbent, *V* (L) is the volume of the dye solution.

## Figures and Tables

**Table 1:** Iron content, determined by SEM/EDX, and barium content, measured by elemental analysis, for PPy-G aerogel, PPy-G-BaFe aerogels synthesized using various fractions of BaFe, and pristine BaFe.

BaFe Fraction, wt%	SEM/EDX	Elemental Analysis	
	Fe, wt%	Ba, wt%	BaFe_calc_, wt%
0	0	0	0
1%	1.0	0.047 ± 0.001	0.36
2%	0.2	0.075 ± 0.002	0.57
5%	0.8	0.13 ± 0.02	1.02
10%	0.7	0.51 ± 0.03	3.90
Pristine BaFe	18.5	13.1 ± 0.4	100

**Table 2:** Mechanical properties (cryogels), conductivity and specific surface area of PPy-G aerogel and PPy-G-BaFe aerogels, synthesized using various fractions of BaFe.

BaFe Fraction, wt%	Tensile Strain at Break, %	Tensile Stress at Break, kPa	Tensile Modulus, kPa	Conductivity, S cm^−1^	BET Surface Area, m^2^ g^−1^
0	7 ± 1	12 ± 3	321 ± 24	1 × 10^−5^	7.3
1%	7 ± 1	10 ± 3	267 ± 54	2 × 10^−4^	11.0
2%	6 ± 1	8 ± 1	289 ± 42	3 × 10^−4^	12.7
5%	6 ± 2	6 ± 1	247 ± 53	4 × 10^−4^	18.3
10%	6 ± 2	7 ± 4	299 ± 42	6 × 10^−5^	19.3

**Table 3:** Fitted kinetics parameters and experimental *Q_e_* of Reactive Black 5 adsorption kinetics with PPy-G-BaFe (10 wt%) and PPy-G as adsorbents.

Material/Mass	*Q_e_* (exp), mg g^−1^	Pseudo-First-Order			Pseudo-Second-Order			Intraparticle Diffusion		
		*k_1_*, min^−1^	*Q_e_*, mg g^−1^	*R^2^*	*k_2_*, g mg^−1^ min^−1^	*Q_e_*, mg g^−1^	*R^2^*	*k_i_*, mg g^−1^ min^−1/2^	*c*, mg g^−1^	*R^2^*
PPy-G/52 mg	42.9	0.0005	35.3	0.975	0.00016	45.5	0.991	1.338	3.6	0.937
PPy-G-BaFe (10 wt%)/52 mg	47.9	0.0009	39.4	0.984	0.00022	51.6	0.999	non-linear		
PPy-G-BaFe (10 wt%)/26 mg	89.5	0.0002	69.3	0.959	3 × 10^−5^	92.5	0.991	1.478	10.8	0.909
PPy-G-BaFe (10 wt%)/104 mg	23.9	0.0031	23.9	0.988	0.0014	24.9	0.998	non-linear		

## Data Availability

Data will be available upon request.

## Supplementary Materials

The following supporting information can be downloaded at: https://www.mdpi.com/article/10.3390/gels9020092/s1, Figure S1. SEM/BSE images of PPy-G-BaFe aerogels, synthesized using (a) 1 wt% of BaFe, (b) 2 wt% of BaFe and (c) 5 wt% of BaFe. Figure S2: Peak deconvolution of the amorphous peak in PPy-G aerogel spectrum in comparison to the spectrum of pristine PPy; Figure S3: Details of the XRD patterns of PPy-G-BaFe aerogels, prepared using various fractions of BaFe, and pristine BaFe from 25 to 45 degrees, highlighting the shift of BaFe peaks to lower angles. Figure S4. Evolution of UV-vis spectra of Reactive Black 5 solution (50 mg l^−1^) over time during the adsorption studies with (a) 52 mg of PPy-G aerogel, (b) 26 mg of PPy-G-BaFe aerogel, prepared at 10 wt% BaFe fraction (c) 52 mg of PPy-G-BaFe aerogel, prepared at 10 wt% BaFe fraction, and (d) 104 mg of PPy-G-BaFe aerogel, prepared at 10 wt% BaFe fraction. Table S1. Adsorption capacity of various materials towards Reactive Black 5.
