# Supplementary material for: Polypyrrole-Barium Ferrite Magnetic Cryogels for Water Purification
Source: Gels. 2023 Jan 20;9(2):92. doi: 10.3390/gels9020092 (PMC9957020; doi:10.3390/gels9020092)
Supplement: Supplementary file 1 [file gels-09-00092-s001.zip › gels-2149354-supplementary.pdf]

gels

MDPI

Supplementary Materials

Polypyrrole-Barium Ferrite Magnetic Cryogels for Water Purification

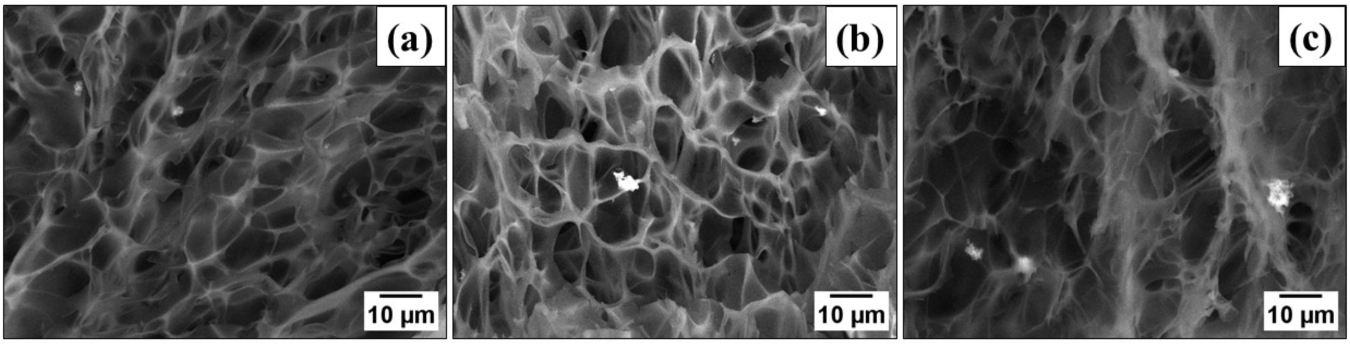

Figure S1. SEM/BSE images of PPy-G-BaFe aerogels, synthesized using (a) 1 wt% of BaFe, (b) 2 wt% of BaFe and (c) 5 wt% of BaFe.

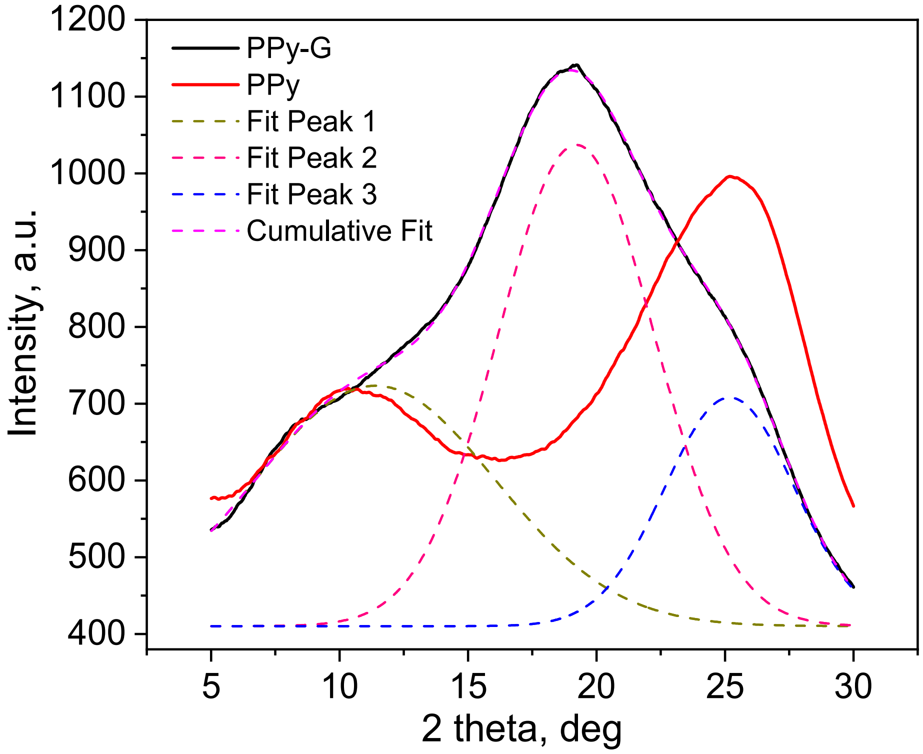

Figure S2. Peak deconvolution of the amorphous peak in PPy-G aerogel spectrum in comparison to the spectrum of pristine PPy.

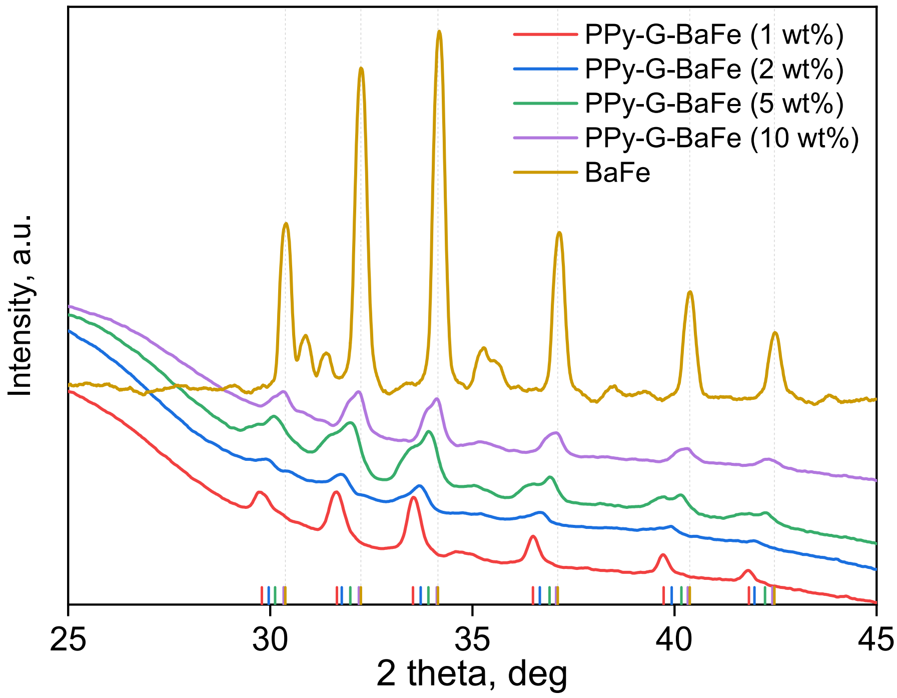

Figure S3. Detail of the XRD patterns of PPy-G-BaFe aerogels, prepared using various fractions of BaFe, and pristine BaFe from 25 to 45 degrees, highlighting the shift of BaFe peaks to lower angles.

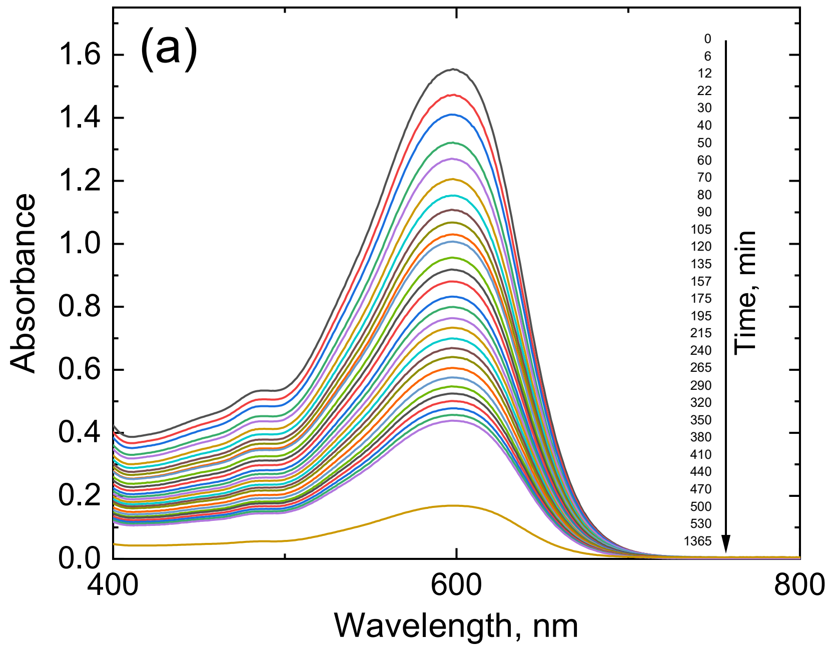

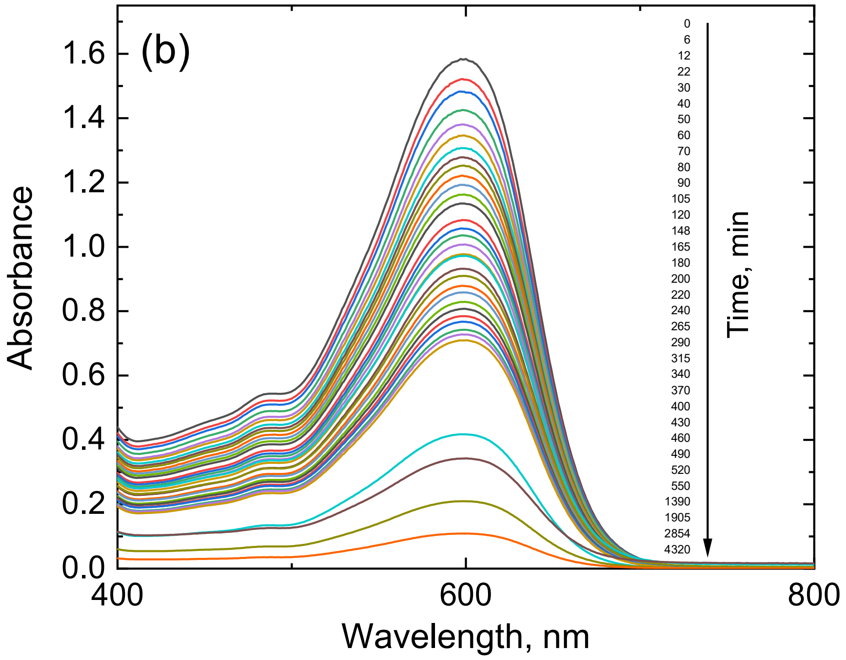
(b)
Absorbance
Wavelength, nm
Time, min
0.0
0.2
0.4
0.6
0.8
1.0
1.2
1.4
1.6
400
600
800
0
6
12
22
30
40
50
60
70
80
90
105
120
148
165
180
200
220
240
265
290
315
340
370
400
430
460
490
520
550
1390
1905
2854
4320

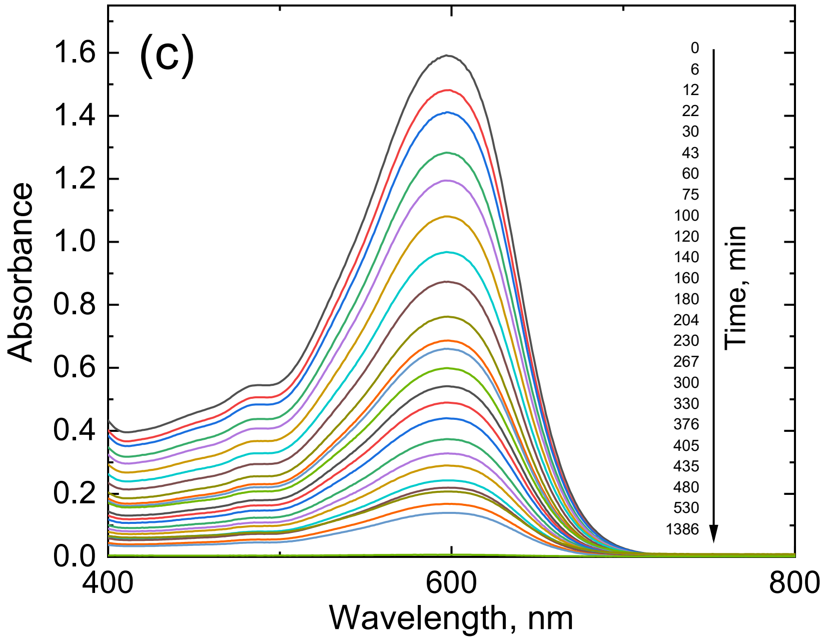
(c)
Absorbance
Wavelength, nm
Time, min
0.0
0.2
0.4
0.6
0.8
1.0
1.2
1.4
1.6
400
600
800
0
6
12
22
30
43
60
75
100
120
140
160
180
204
230
267
300
330
376
405
435
480
530
1386

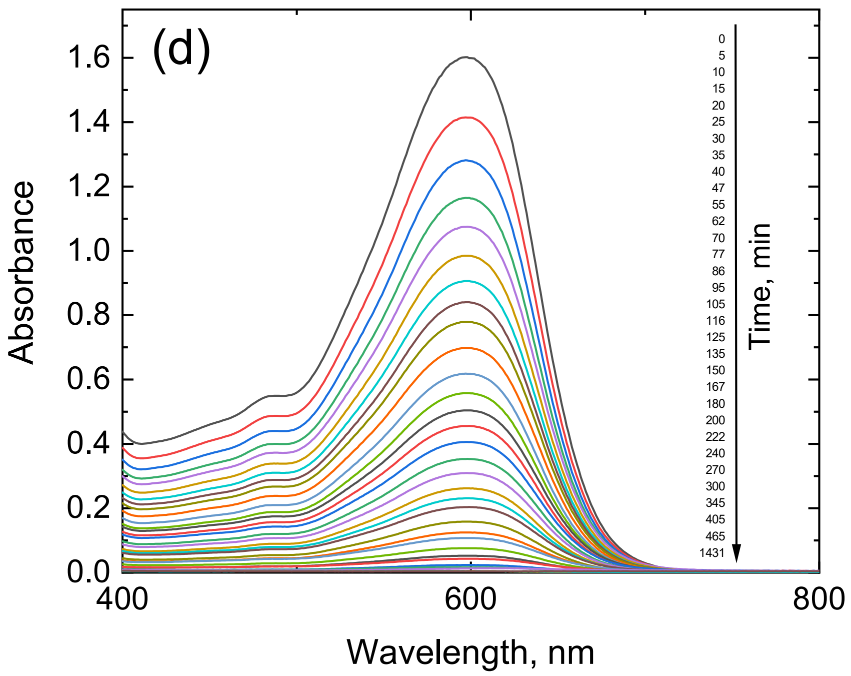

Figure S4. Evolution of UV-vis spectra of Reactive Black 5 solution (50 mg l^{-1}) over time during the adsoprtion studies with (**a**) 52 mg of PPy-G aerogel, (**b**) 26 mg of PPy-G-BaFe aerogel, prepared at 10 wt% BaFe fraction (**c**) 52 mg of PPy-G-BaFe aerogel, prepared at 10 wt% BaFe fraction, and (**d**) 104 mg of PPy-G-BaFe aerogel, prepared at 10 wt% BaFe fraction.

Table S1. Adsorption capacity of various materials towards Reactive Black 5.

Material	Adsorption capacity, mg g^{-1}	Reference
Powdered activated carbon	58.8	[1]
Walnut activated carbon	19.3	[2]
Polyhydroxyl dendrimer magnetic nanoparticles	62.1	[3]
CTAB-modified zeolite	15.9	[4]
Poly(p-phenylenediamine) / maghemite	233	[5]
Poly(3,4-ethylenedioxythiophene) / maghemite	60	[6]
Polypyrrole / zeolite	122.3	[7]
Polyaniline / magnetite	63.7	[8]

References

1. Eren, Z.; Acar, F.N. Adsorption of Reactive Black 5 from an aqueous solution: Equilibrium and kinetic studies. *Desalination* **2006**, *194*, 1–10. https://doi.org/10.1016/j.desal.2005.10.022.
2. Heibati, B.; Rodriguez-Couto, S.; Amrane, A.; Rafatullah, M.; Hawari, A.; Al-Ghouti, M.A. Uptake of Reactive Black 5 by pumice and walnut activated carbon: Chemistry and adsorption mechanisms. J. Ind. Eng. Chem. **2014**, *20*, 2939–2947. https://doi.org/10.1016/j.jiec.2013.10.063.
3. Galangash, M.M.; Ghavidast, A.; Bozorgpanah, Z. Adsorption of acid red 114 and reactive black 5 in aqueous solutions on dendrimer-conjugated magnetic nanoparticles. *J. Chin. Chem. Soc*. **2019**, *66*, 62–74. https://doi.org/10.1002/jccs.201800177.
4. Karadag, D.; Turan, M.; Akgul, E.; Tok, S.; Faki, A. Adsorption equilibrium and kinetics of reactive black 5 and reactive red 239 in aqueous solution onto surfactant-modified zeolite. *J. Chem. Eng. Data* **2007**, *52*, 1615–1620. https://doi.org/10.1021/je7000057.
5. Minisy, I.M.; Zasońska, B.A.; Petrovský, E.; Veverka, P.; Šeděnková, I.; Hromádková, J.; Bober, P. (Poly(p-phenylenediamine)/maghemite composite as highly effective adsorbent for anionic dye removal. *React. Funct. Polym*. **2020**, *146*, 104436. https://doi.org/10.1016/j.reactfunctpolym.2019.104436.
6. Gupta, S.; Zasońska, B.A.; Acharya, U.; Konefał, M.; Pokorný, V.; Petrovsky, E.; Breitenbach, S.; Unterweger, C.; Bober, P. Magnetoconductive poly(3,4-ethylenedioxythiophene)/maghemite adsorbent for the removal of Reactive Black 5 from aqueous media. *Mater. Chem. Phys*. **2022**, *292*, 126753. https://doi.org/j.matchemphys.2022.126753.
7. Senguttuvan, S.; Janaki, V.; Senthilkumar, P.; Kamala-Kannan, S. Polypyrrole/zeolite composite - A nanoadsorbent for reactive dyes removal from synthetic solution. Chemosphere **2022**, *287*, 132164. https://doi.org/10.1016/j.chemosphere.2021.132164.
8. Hamzehloo, M.; Farahani, B.K.A.; Rostamian, R. Adsorption Behaviour of Reactive Black Dye 5 by Magnetically Separable Nanoadsorbent. *Phys. Chem. Res*. **2019**, *7*, 475–490. https://doi.org/10.22036/pcr.2019.186198.1639.
